# Reduced nitric oxide levels during drought stress promote drought tolerance in barley and is associated with elevated polyamine biosynthesis

**DOI:** 10.1038/s41598-017-13458-1

**Published:** 2017-10-17

**Authors:** Gracia Montilla-Bascón, Diego Rubiales, Kim H. Hebelstrup, Julien Mandon, Frans J. M. Harren, Simona M. Cristescu, Luis A. J. Mur, Elena Prats

**Affiliations:** 1 CSIC, Institute for Sustainable Agriculture, Córdoba, Spain; 2 Section of Crop Genetics and Biotechnology, Department of Molecular Biology and Genetics Aarhus University, Slagelse, Denmark; 3 Radboud University, Department of Molecular and Laser Physics, Nijmegen, The Netherlands; 4 Institute of Biological, Environmental and Rural Sciences, University of Aberystwyth, Aberystwyth, UK

## Abstract

Nitric oxide (NO) is a key messenger in plant stress responses but its exact role in drought response remains unclear. To investigate the role of NO in drought response we employed transgenic barley plants (UHb) overexpressing the barley non-symbiotic hemoglobin gene HvHb1 that oxidizes NO to NO_3_^−^. Reduced NO production under drought conditions in UHb plants was associated with increased drought tolerance. Since NO biosynthesis has been related to polyamine metabolism, we investigated whether the observed drought-related NO changes could involve polyamine pathway. UHb plants showed increases in total polyamines and in particular polyamines such as spermidine. These increases correlated with the accumulation of the amino acid precursors of polyamines and with the expression of specific polyamine biosynthesis genes. This suggests a potential interplay between NO and polyamine biosynthesis during drought response. Since ethylene has been linked to NO signaling and it is also related to polyamine metabolism, we explored this connection. *In vivo* ethylene measurement showed that UHb plants significantly decrease ethylene production and expression of aminocyclopropane-1-carboxylic acid synthase gene, the first committed step in ethylene biosynthesis compared with wild type. These data suggest a NO-ethylene influenced regulatory node in polyamine biosynthesis linked to drought tolerance/susceptibility in barley.

## Introduction

Drought is considered the most important stress contributing to yield and economical losses in many regions worldwide^1^. Understanding plant tolerance to drought is therefore of fundamental importance and represents a major topic of research. Drought, as with other environmental stresses, elicits profound changes in the plants at gene, protein and metabolite levels as they either succumb to its effects and/or deploy tolerance mechanisms. The coordination of such changes via signaling events is of immense interest and over the years a number of key molecules have been defined; most particularly abscisic acid (ABA)^2^.

Over the last decade the free radical signal nitric oxide (NO) has been linked with an increasing number of signaling pathways controlling processes that range from biotic and abiotic stress responses to growth and development (reviewed in^3,4^). The regulation of the stomatal aperture by NO is located at the crossroads between developmental and abiotic stress responses^3,5^. During the induction of stomatal closure, ABA induces NO generation together with an increase in cytoplasmic pH and H_2_O_2_^6,7^. Accordingly, stomatal closure elicited by exogenously applied NO donors (i.e. sodium nitroprusside, SNP) conferred tolerance to rapid dehydration in wheat seedling^8^. However, NO can be a redundant element under conditions of rapid dehydration^9^. Given this ambiguity, it is somewhat surprising that the patterns of *in vivo* NO generation in intact plants undergoing water stress has not been determined as a preliminary to investigate the possible roles of NO during drought.

Polyamines are low molecular weight nitrogenous metabolites considered to be ubiquitous in all living cells. These molecules are positively charged at physiological pH and hence, initially, their biological function was associated with their capability of binding to negatively charged molecules, such as nucleic acids, phospholipids, and proteins. These ionic interactions, which are reversible, lead to the stabilization of DNA, RNA, membranes and some proteins^10,11^. In addition to stabilizing macromolecular structures, polyamines also act as hormones or regulatory molecules in many fundamental cellular processes as well as in senescence and stress responses^12,13^. The di-amine Putrescine (Put), the tri-amine spermidine (Spd) and tetra-amine spermine (Spm) are the most common polyamines in plants, although agmatine, cadaverine and thermospermine are also found.

**Figure 1:**
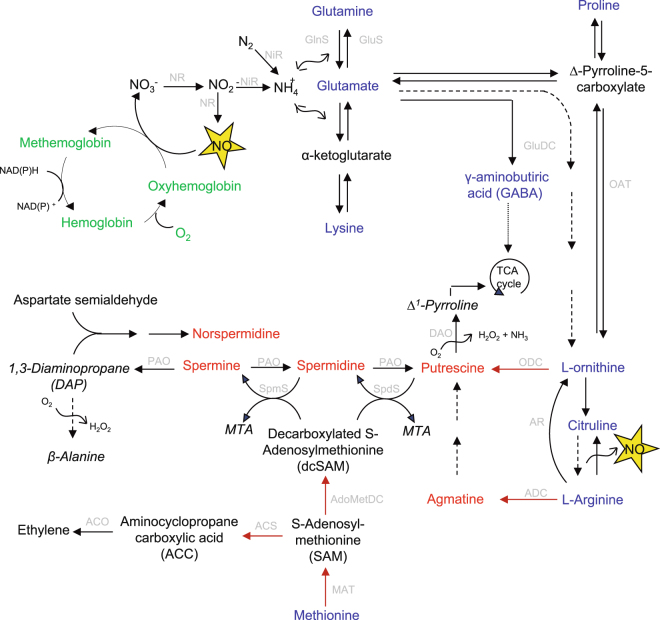
Polyamine metabolism and interaction with other metabolic routes modified from Alcazar *et al*., 2010 and Imgarberdiev *et al*., 2011. Red names indicate polyamines, blue names indicate aminoacids, green names indicate the simplified haemoglobin cycle, grey abbreviations correspond to enzymes: NR, nitrate reductase; NIR: nitrite reductase; GlnS, glutamine synthetase; GluS: glutamate synthase; GluDC: glutamate decarboxylase; OAT: ornithine δ-aminotransferase; AR: Arginase; ODC: ornithine decarboxylase; ADC: arginine decarboxylase; MAT: synthetase, AdoMetDC: decarboxylase; SpmS spermine synthase, SpdS spermidine synthase; DAO: diamine oxidase: PAO: polyamine oxidase, ACS: ACC synthase; ACO: ACC oxidase. It has been shown recently that polyamine oxidase is not only involved in the terminal catabolism of polyamines, but also in the back-conversion of spermine to spermidine and spermidine to putrescine.

NO might interact with polyamines during developmental and stress responses. For example, NO production has been observed in response to exogenously applied polyamines^14^.This is of particular relevance to drought, as a protective role for specific polyamines against drought stress has been reported in Arabidopsis. For instance, Spm by modulating the activity of certain ion channels, increases Ca^2+^, thus regulating stomatal closure^15^. In cereals, changes in polyamines contents have also been reported during drought stress^16,17^. Furthermore, several amino acids, which are direct precursors of polyamines also influence nitric oxide synthesis^18^ (Fig. 1). Indeed, in mammalian cells^19^ and plants^20^, arginases were reported to play an important role in directing the metabolism of L-arginine to either polyamines or NO. However, the mechanisms governing the relationship between NO and polyamines is currently obscure. It is unclear whether polyamines act as substrates, cofactors, or signals for promoting NO synthesis^21^. In addition, whereas a possible effect of NO on polyamine biosynthesis has been reported in some studies^22^ this was not the case in others^23^.

Nitric oxide content in the cell not only depends on its biosynthesis but also on its removal from the cell. NO can be eliminated by oxidation to NO_3_ following the formation of an oxidized form of hemoglobin (methemoglobin), from which the reduced form may be regenerated by monodehydroascorbate reductase^24^ (Fig. 1). As such, plant hemoglobins (Hb) are well known modulators of NO in plants^25,26^. There are three classes of plant hemoglobins designated class 1, 2 and 3. Plant hemoglobins of the class 1 type have earlier been demonstrated to be effective modulators of NO mediated stress responses, such as the responses to flooding and hypoxia in Arabidopsis^27^ as well as in barley^28^ and in NO mediated responses to pathogens^3^. Barley type 1 Hb is an effective scavenger of endogenous NO. Thus, barley plants overexpressing the Hb gene had a significantly lower NO emission than wild type (WT) plants, with the reduction in NO levels directly attributed to Hb gene and protein expression^28^. The effect was equivalent to previous observations in Arabidopsis^27^ demonstrating that barley class 1 Hb is as efficient as Arabidopsis class 1 Hb in scavenging NO.

To investigate drought stress responses we used these transgenic barley lines (UHb) overexpressing the class 1 barley hemoglobin gene HvHb1. Our work shows that HvHb1-over expression decreased NO levels and this conferred increased drought tolerance. Molecular characterization of the WT and UHb lines suggested that a reduced NO production resulted in subtle reprogramming of polyamine biosynthesis and associated gene-expression. It also reduced expression of ethylene biosynthetic genes and ethylene emission which was linked to delayed senescence related symptoms and drought tolerance.

## Results

### UHb barley plants showed reduced levels of NO compared with WT

**Figure 2:**
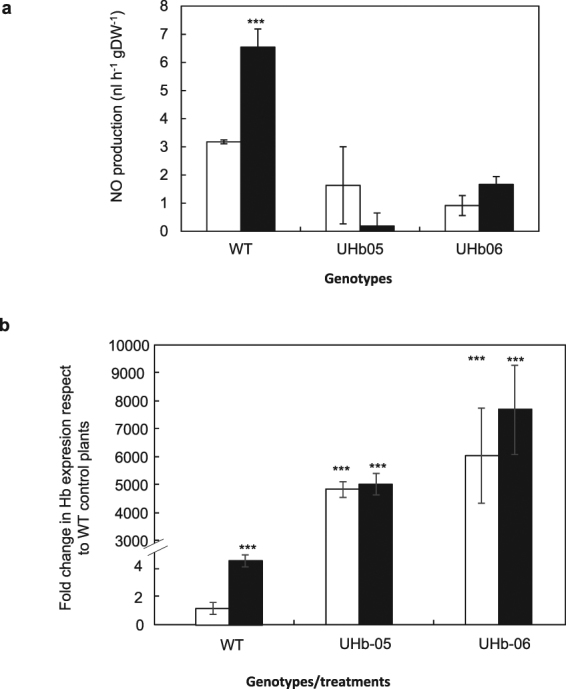
*In vivo* NO generation and hemoglobin gene expression. (**a**) *In vivo* NO measurement in barley UHb-05 and UHb-06 lines and WT Golden Promise intact plants. (**b**) Expression of HvHb1 gene. White bar = watered controls; Black bars = plants exposed to drought treatment. Data are mean of four replicates ± standard error. *, **, *** indicate significant differences at P < 0.05, 0.01 and 0.001, respectively with respect to WT control plants.

In order to dissect the role of NO during water stress, two different HvHb1 overexpressing barley lines (UHb) were assessed. Quantum cascade laser (QCL) spectroscopy was used to assess whether NO production was reduced in both UHb lines, UHb-05 and UHb-06, compared to WT (Golden Promise) in response to water stress. As expected^28^, under well-watered conditions, UHb plants showed significantly reduced levels of NO compared to those in the WT (Fig. 2A). With water stress, WT plants NO production increased more than two fold compared to controls whereas UHb plants showed no significant differences in NO levels (Fig. 2A). This confirmed the efficacy of the barley UHb lines in scavenging significant amounts of the NO generated by the plant. A significant 4-fold overexpression of Hb gene compared to WT well-watered controls was observed in WT plants under drought. However, as expected, in UHb-05 and UHb-06 genotypes, Hb gene was dramatically over expressed under well-watered conditions and this expression was maintained under drought (Fig. 2B).

### UHb barley plants showed increased drought tolerance compared with WT

**Figure 3:**
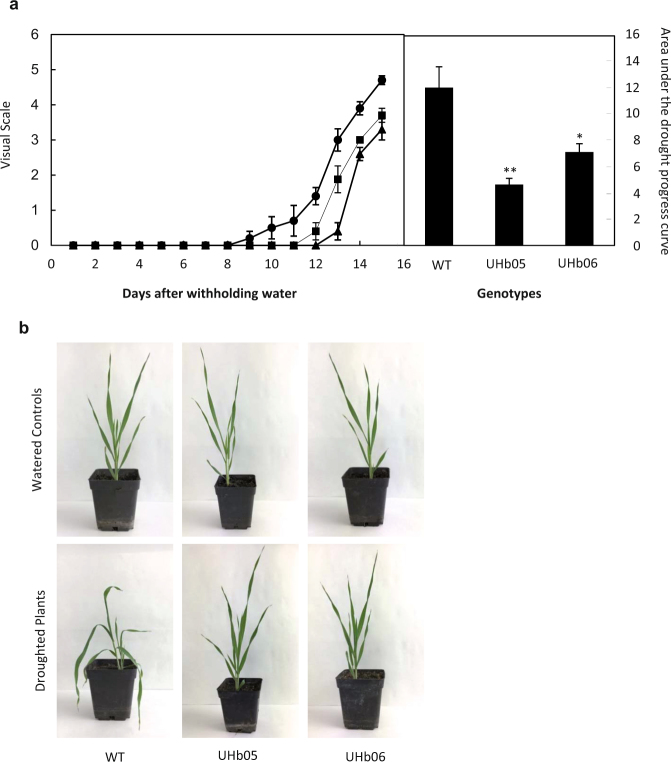
Drought stress symptoms in UHb and wild type (WT) Golden Promise plants. (**a**) Drought symptoms were evaluated after withholding water according to a visual scale (Sánchez-Martín *et al*.^33^) in UHb-05 (triangles), UHb-06 (squares) and WT (circles) plants. Bars in the right panel represent the area under the drought progress curve to assess quantitative stress tolerance, (for details see^57^). Data are mean of five replicates ± standard error. *, ** indicate significant differences at P < 0.05 and 0.01, respectively between WT and UHb plants. (**b**) Pictures of WT and UHb plants at 20–25% of sRWC showing more drought symptoms in WT plants.

To determine the effect of NO reduction in drought stress symptoms, WT and UHb plants were evaluated during a water stress time course. To ensure accurate comparison between WT and UHb plants, the relative water content in the soil (sRWC) was monitored daily during experiments showing that both genotypes followed a similar sRWC curve (Supplementary Fig. 1). Wild type plants started to exhibit visual symptoms of drought 8 days after withholding water (daww) but in UHb lines these were seen from 11-12 daww (Fig. 3A,B). The area under the drought progress curve was significantly (*P* = 0.003) and greatly reduced, between 2 and 3-fold, in UHb-05 and UHb-06, respectively, compared with the WT (Fig. 3A).

**Figure 4:**
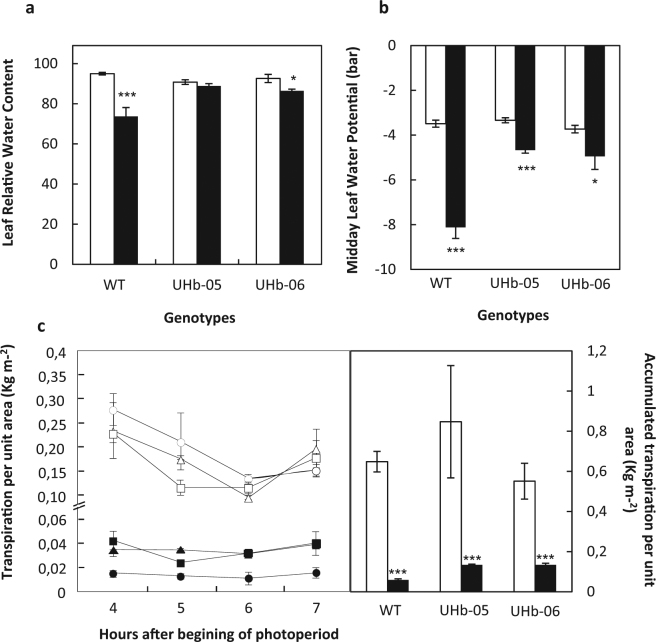
Water related parameters in UHb and wild type (WT) Golden Promise plants. (**a**) Leaf relative water content. (**b**) Midday water potential, and (**c**) Transpiration. Transpiration rate per unit area was measured at different sampling times (left panel) and as accumulated transpiration per unit area (right panel) representing the sum of all transpired water over the assessed period. All parameters were evaluated at 20–25% soil relative water content in UHb-05 (triangles), UHb-06 (squares), and WT (circles) plants. White bars/symbols = control, well-watered plants; Black bars/symbols = plants exposed to water stress. Data are mean of five replicates ± standard error. *, *** indicate significant differences between droughted and well-watered plants at P < 0.05, and 0.001 respectively.

To confirm the increased drought tolerance phenotype of the UHb plants, several physiological parameters related to water balance were measured in barley WT and UHb plants (Fig. 4). All genotypes showed reduced levels of leaf relative water content under water stress compared to their respective well-watered controls (Fig. 4A). However, UHb genotypes showed a significant higher leaf RWC than the wild type plants under drought (*P* < 0.001 and *P* = 0.002, respectively for UHb-05 and UHb-06). No differences in the leaf relative water content of well-watered plants between genotypes were observed. Assessment of the midday leaf water potential showed a similar trend than with leaf RWC albeit differences between WT and UHb plants were clearer. In all genotypes the water potential under drought stress was significantly reduced with respect to watered controls but whereas WT plants doubled the negative water potential compared to controls, UHb plants only showed a slight reduction. Thus, droughted WT plants showed a significantly higher negative water potential than droughted UHb plants (*P* < 0.001; Fig. 4B). When transpiration was measured during the central part of photoperiod no differences were seen between well-watered controls but under water stress there was a slight but significant increase in transpiration in UHb plants compared to WT plants (*P* < 0.001 for both UHb genotypes) (Fig. 4C).

### Specific polyamines increased in UHb lines compared to WT under drought

**Figure 5:**
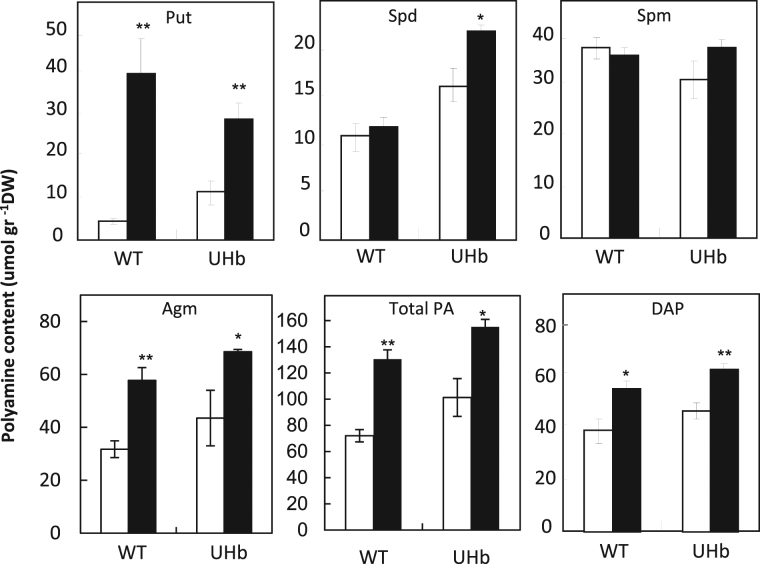
Polyamine content in UHb and wild type (WT) Golden Promise plants. Putrescine, spermidine, spermine, agmatine, their total content (total PA) and the degradation product, 1,3-diamino-propane (DAP) were measured at 20% soil relative water content in UHb and WT plants. White bars = control, well-watered plants; Black bars = plants exposed to drought treatment. Data are mean of five replicates ± standard error. *, **, indicate significant differences between droughted and well-watered plants at P < 0.05, and 0.01 respectively.

NO biosynthesis is closely related to polyamine and some amino acid metabolic pathways (Fig. 1). Furthermore, a possible linkage between polyamines and NO-mediated effects has been reported^14^. Thus, we measured polyamine content in UHb and WT plants to determine if these could be correlated with the increased tolerance to drought observed in the UHb plants. Since both, UHb-05 and 06 lines showed (for example) similarly reduced NO levels and hemoglobin expression, this was performed in UHb-05 plants. UHb plants showed higher constitutive levels of Put, and Spd than WT plants (*P < *0.05; Fig. 5). On the application of water stress, the content of these polyamines significantly increased in both genotypes. The levels of Put increased in both the WT and UHb plants but significantly less so with the latter (Fig. 5). By contrary, Spd did not significantly increase in WT plants following drought stress whereas it increased in UHb plants (p < 0.05). Thus, the content of Spd in UHb plants under drought stress was near 2-fold the content of WT plants. Spm did not significantly change in either genotype and reached similar levels under drought. Increases of agmatine and DAP were also observed in both WT and UHb plants subjected to drought with slightly higher levels in UHb plants (Fig. 5). Overall, total PA content significantly increased in both WT and UHb droughted plants compared with their respective well-watered controls. However under both, well-watered conditions and drought, UHb plants showed higher total PA content compared with their corresponding WT plants (*P < *0.05).

### Levels of amino acids linked to the polyamine pathway differed between UHb and WT plants

**Figure 6:**
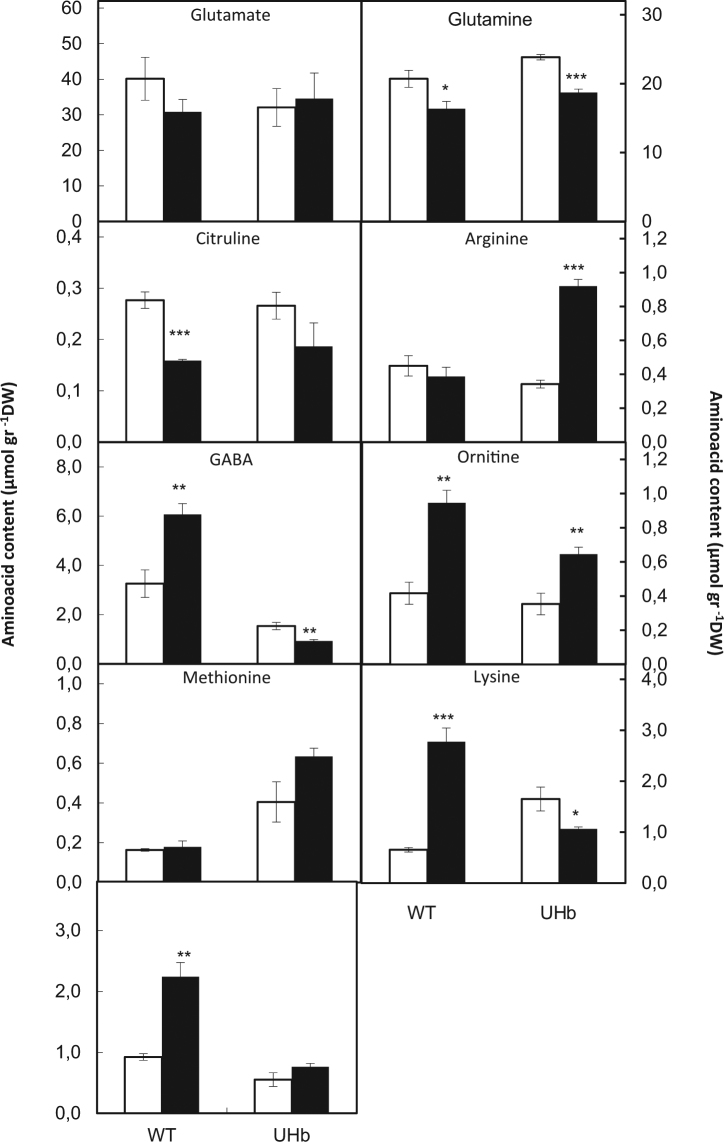
Amino acids content in UHb and wild type (WT) Golden Promise plants. Amino acids related with polyamine biosynthesis pathway were measured at 20% soil relative water content in UHb and WT plants. White bars = control, well-watered plants; Black bars = plants exposed to drought treatment. Data are mean of five replicates ± standard error. *, **, *** indicate significant differences between droughted and well-watered plants at P < 0.05, 0.01 and 0.001 respectively.

As NO generation has been intimately linked with nitrate reductase activity (e.g. Gupta *et al*.,^29^, Fig. 1), changes in polyamine biosynthesis could be reflecting wider changes in nitrogen assimilation/amino acid production^30^. Furthermore, several amino acids are at the crossroad between polyamine and NO biosynthesis (Fig. 1.). To investigate this, the concentrations of a range of amino acids directly linked to the polyamine pathway in UHb and WT plants were determined (Fig. 6).

For several of the amino acids, both WT and UHb plants followed a similar trend under water stress. Thus, in both genotypes water stress resulted in decreased levels of glutamine but increased levels of ornithine, the latter slightly higher in the WT. However, a quite different trend was observed for key amino acids of the polyamine pathway such as arginine, γ-aminobutyric acid (GABA), methionine, lysine and proline (Fig. 6). UHb plants showed a significant increase in arginine and methionine particularly under drought stress compared with WT plants. By contrast, WT showed a significant increase in GABA, lysine and proline under drought stress with respect to UHb plants (Fig. 6). These results suggest a role for NO in directing biosynthetic pathways away from the polyamine pathway.

**Figure 7:**
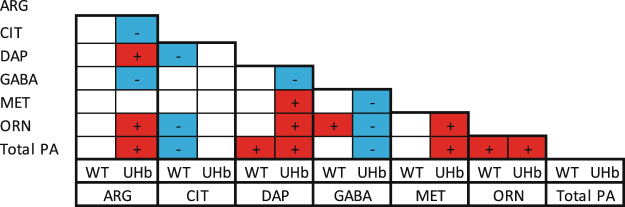
Scheme of Pearson correlations between total polyamines (PA) and their most direct amino acids precursors (Arginine, ARG; Citrulline, CIT; Methionine, MET; Ornithine, ORN) and derivatives (ϒ-aminobutiric acid, GABA; and 1,3-diaminopropane, DAP) in barley WT and UHb plants. +/ red-colored squares indicated significant positive correlations and −/blue-colored squares indicate significant negative correlations. More details about the correlation coefficients and significance can be accessed in Supplementary Table 1.

This role of NO in drought response was also indicated by several significant correlations found between polyamines and amino acids (Fig. 7, Supplementary Table 1). In UHb plants, total PA content positively correlated with arginine, ornithine and methionine, the most direct PA precursors, suggesting that reduced NO might favour an increase in these amino acids, and PA biosynthesis (as observed). In WT plants total PA content was only significantly correlated with ornithine, whilst this amino acid positively correlated with GABA content. This, together with the observed increase in GABA under drought in WT plants, suggests that the PA biosynthetic flux could be biased to GABA and TCA cycle instead to spermidine or spermine most likely via the GABA shunt mechanism (Fig. 1). Interestingly and following an inverse trend, in UHb plants ornithine but also arginine, total PA and methionine were negatively correlated with GABA (Fig. 7). Furthermore, in UHb plants citrulline, which is generated during the conversion of arginine to NO, was negatively correlated with arginine. This together with the increases in arginine observed under drought in UHb plants suggest a diversion of the arginine pool away from NO and citrulline production.

### NO regulates the expression of several polyamine biosynthesis genes

**Figure 8:**
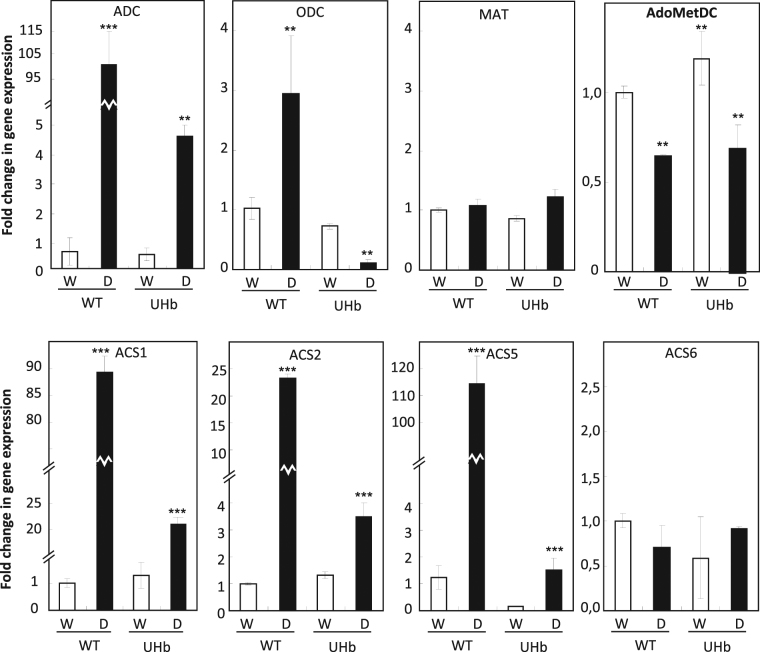
Expression of several genes involved in the polyamine pathway in UHb and wild type (WT) Golden Promise plants. Expression of arginine decarboxylase (ADC), ornithine decarboxylase (ODC), methionine adenosyltransferase (MAT), S-Adenosylmethionine decarboxylase (AdoMetDC), and 1-aminocyclopropane-1-carboxylate [ACC] synthase (ACS) were measured at 20% soil relative water content in UHb and WT plants. White bars = control, well-watered plants (W); Black bars = plants exposed to drought treatment (D). Data, expressed as fold change in expression respect to WT well-watered plants, are mean of at least 3 independent biological plus 3 technical replications ± standard error. *, **, *** indicate significant differences with respect WT well-watered plants at P < 0.05, 0.01 and 0.001, respectively.

We next investigated whether NO might influence polyamine levels through regulation of key genes of their biosynthesis pathway. Thus, quantitative RT-PCR experiments were carried out to determine the expression of key enzymes of the polyamine pathway, arginine decarboxylase (ADC), ornithine decarboxylase (ODC), methionine adenosyltransferase (MAT), S-Adenosylmethionine decarboxylase (AdoMetDC) and genes of the linked ethylene biosynthetic pathway; 1-aminocyclopropane-1-carboxylate [ACC] synthase (ACS) (Fig. 1) of which 4 different isoforms have been described in barley. Expression of ADC increased significantly in both, WT and UHb plants under drought stress. However, the increase was substantially and significantly higher (*P* < 0.001) in the WT compared to UHb plants (Fig. 8). No significant differences were observed between genotypes in well-watered conditions. The level of the expression of ADC was significant (*P* = 0.003) and positively (r^2^ = 0.87) correlated with the level of drought symptoms (AUDPC) observed in the plants. This correlation between gene expression and drought symptoms was also observed when assessing *ACS5*.

ODC expression followed a different trend in WT and UHb plants. Thus, whereas ODC expression up-regulated in WT plants it was down-regulated in UHb lines. No significant differences were observed in MAT expression in WT and UHb plants. Interestingly, a significant (*P < *0.01) down-regulation of AdoMetDC was observed in WT and UHb plants subjected to drought stress. Finally, a major up-regulation of the ethylene biosynthetic *ACS* genes, *ACS1*, *ACS2* and *ACS5*, of up to more than 100-fold was observed in WT plants subjected to water stress, whereas only slightly higher increases in the expression levels (3.5-fold) were observed in UHb plants. No significant differences in ACS6 expression were observed between genotypes (Fig. 8).

### UHb barley plants showed reduced levels of ethylene compared with WT

**Figure 9:**
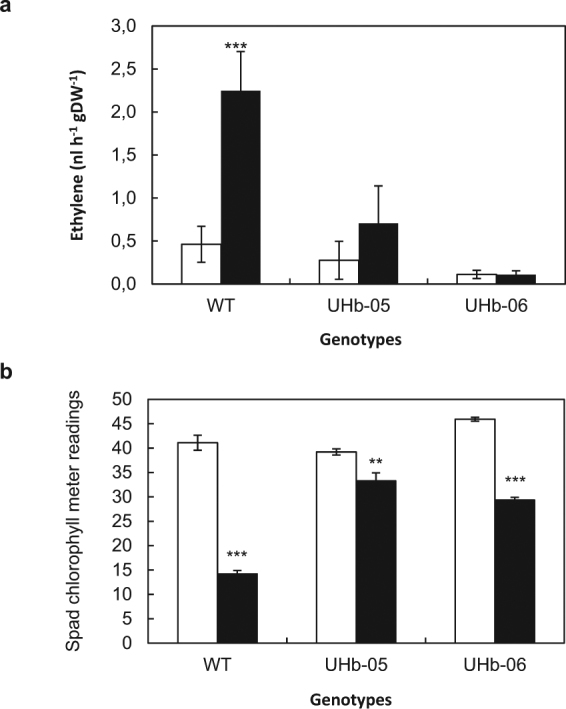
Ethylene generation and senescence symptoms in UHb and WT Golden Promise plants. (**a**). *In vivo* ethylene generation in barley UHb plants and WT Golden Promise. Ethylene was quantified in whole intact UHb and wild type plants at 20–25% soil relative water content. Data are mean of four replicates + standard error. (**b**). Spad chlorophyll meter readings (SCMR) of barley UHb plants and WT Golden Promise. SCMR were assessed in the first leaf at 20–25% sRWC. Data are based on five replicates per genotype and treatment and three SCMR per leaf. White bar = watered controls (W); Black bars = plants exposed to drought treatment (D). **, *** indicate significant differences at P < 0.01, and 0.001 with respect to control plants.

The higher levels of ACS gene in WT plants under drought compared to UHb plants suggested an NO-mediated diversion of metabolites from polyamine biosynthesis to ethylene pathway in susceptible plants. To investigate this, *in vivo* ethylene emission was measured in WT and UHb intact plants under well-watered and drought conditions. Ethylene production followed a similar pattern to that observed for NO, with WT plants showing an increase of ethylene under water stress (*P* < 0.001). No significant differences in ethylene production were observed in UHb-05 and UHb-06 plants under drought compared to their well-watered controls (Fig. 9A).

Ethylene is related to stress-induced leaf senescence and abscission^31^, thus we estimated leaf chlorophyll through a SPAD chlorophyll meter. Data from Fig. 9B shows that WT plants had lower chlorophyll content than both UHb lines under drought (*P* < 0.001) confirming the visual yellowing symptoms observed in older leaves of WT plants under drought (Fig. 3B), indicating drought-induced senescence and hence less drought tolerance.

## Discussion

Our work shows that the transgenic UHb barley plants, overexpress the HvHB1 both under well-watered and drought conditions, showing reduced NO levels and increased drought tolerance compared to the WT plants. Previous work by^8^ reported an effect of NO conferring water deficit tolerance when it was exogenously applied using SNP. These authors showed that 150 µM SNP increased the RWC of detached wheat leaves subjected to different periods of drought for no longer than 3 h. The higher RWC of SNP-treated leaves was associated to a decrease in the water loss achieved by stomata closure. The differences observed between that study and our study with respect to the effect of NO in drought tolerance might be explained by different reasons: In the conditions of the detached leaves, closure of stomata would be crucial for maintaining the leaf RWC, and the NO supplied by the SNP could contribute to this, since it is known that ABA induced NO generation results in stomatal closure^6,32^. However, as it has been previously reported^33,34^ at whole plant level, maintenance of water balance is much complex and fine modulation of stomata closure, more than rapid and tight closure seems to contribute to drought tolerance^33^. Such a feature is likely to contribute to the maintenance of higher root hydraulic conductance and therefore better water status and lower oxidative stress. This was observed in UHb plants, which showed higher drought tolerance (i.e. higher RWC and lower leaf water potential) but maintained slight but significant higher transpiration than the WT plants. The higher transpiration rate might be a direct consequence of the lower NO levels resulting in increased stomatal apertures, which are known to be regulated by NO^6^. On the other hand, there is evidence from Arabidopsis that the effect of NO is concentration-dependent, and that NO when provided in the form of SNP may have opposite effect at concentrations below or above 50 µM^35^. Indeed, it has been also reported that some of the effects observed following SNP treatment can be induced by the interactions of secondary products of NO, so such results need to be considered critically^36^.

Polyamines have most considered to be protective compounds involved in resistance to abiotic and biotic stress^12,37^. Our results suggest a possible involvement of NO in regulating polyamine content as UHb plants with reduced NO levels had increased levels of total polyamines both, constitutively and following drought treatment compared to WT plants. Increase of total polyamines in UHb plants under drought was positively and significantly correlated with increases in the content of several of their direct amino acid precursors, in particular with arginine, methionine and ornithine. In contrast, in WT plants polyamine content was only positively correlated with ornithine and this was correlated with GABA content. This together with the increases observed for these two amino acids and also lysine or proline in WT plants under drought suggest a different fate for N in UHb and WT plants. The different distribution of amino acid content between UHb and WT plants suggest a role for NO diverting the flux of the biosynthetic pathways away from the polyamine pathway, for instance to TCA cycle, lysine and proline, probably through gene expression regulation and/or post-translational modifications. Alternatively, the NO reduction could affect other cellular processes through pleiotropic events that could also mediate in the observed effects.

Overall, WT plants showed also an increase in total polyamine content following water deficit but it was significantly lower than that observed in UHb plants and it was mainly due to a dramatic increase of putrescine that was accompanied by a near 100-fold increase of *ADC* mRNA; far higher than the 5-fold increase observed in the UHb plants. Put levels would be increased in WT plants under drought through *ADC* up-regulation albeit reduced levels of methionine, down-regulation of *AdoMetDC* and up-regulation of *ACS* would reduce its conversion to higher polyamines, Spd and Spm. Previous work^38^ showed that upon the onset of osmotic stress, the ADC protein was post-translationally cleaved to produce a 24 kDa C-terminal fragment containing the ADC active site which promoted putrescine formation^39,40^. Increased levels of Put in the leaves leads to chlorophyll loss and accelerated senescence^41^; features that were also seen in WT plants during water stress. Exogenously applied Spm^41,42^ and Spd^41^ are able to inhibit the post-translational ADC processing thereby decreasing putrescine accumulation and the associated senescence. Our data aligns with these observations and also show a senescence-like phenotype in WT plants under drought which correlated with the higher putrescine accumulation. Our observed shift towards Spd in UHb plants was also supported by Yang *et al*.,^17^ who suggested that high levels of putrescine at an early stage of drought is necessary for plants to adapt to stress by triggering the conversion of putrescine to the higher molecular weight polyamines.

Interestingly, the high levels of Spd observed in UHb plants were not associated with upregulated AdoMetDC or MAT gene. This suggests that Spd in UHb plants could result from further regulation of activities of these enzymes or through putrescine conversion (Fig. 1). Thus, high Spd levels could be being driven by the higher levels of substrate available. Indeed, the aminopropyl groups for Spd or Spm synthesis are ultimately derived from methionine^43^ and methionine content in UHb plants under drought was 3-fold higher than the observed level in WT. Alternatively, AdoMetDC has been described as a target for post-translational inactivation by NO since its activity was lost upon incubation *in vitro* with NO donors such as GSNO and SNAP^44^. This could also explain the lower Spd content observed in WT as this had higher NO levels. Furthermore, the dramatic increase in the *ACS1, ACS2* and *ACS5* transcripts would divert methionine to the ethylene biosynthetic pathway instead to that of polyamines in WT plants (Fig. 1). Indeed, previous studies report an inhibition of ethylene production by polyamines^45^. In agreement with this hypothesis, our data showed a significantly higher increase of the transcript levels of various members of the ACS family in WT plants under drought compared to UHb plants. ACS catalyzes the first committed step in ethylene biosynthesis in higher plants. Thus, the induction of ACS transcription by NO in WT plants under drought, not observed in UHb plants, would greatly contribute to an increase in ethylene accelerating the drought induced senescence in this genotype as has been previously reported in maize^46^. In line with this model, SNP infiltration has been shown to stimulate ACS expression^47,48^ and GSNO stimulate the transcript not only of ACS gene but also other key enzymes involved in ethylene biosynthesis^49^. Our earlier experiments have also demonstrated an effect of class 1 plant Hb silencing and overexpression on ethylene levels in responses to flooding^27^ and biotic stress^3^. In line with these results, *in vivo* measurement of ethylene in the barley intact plants showed increased ethylene emission in WT plants under drought. These data are in agreement with previous work reporting an increase of ethylene and ACS and ACO genes in soybean in response to water stress^50^.In addition, it is now well accepted that ACC, the direct product of ACS, is more than just a precursor of ethylene. The pool of ACC is regulated by a complex interaction of production, consumption, modification and transport^51^. Thus ACC can be conjugated to malonyl-ACC, ϒ-glutamyl-ACC and jasmonyl-ACC, which might serve as a pivotal molecule which can function as a modulator of the hormonal crosstalk between ethylene and jasmonic acid pathway^51^. Furthermore, recent findings have suggested a role for ACC as signaling molecule independent from ethylene^52^.

The complex interaction between NO and ethylene under drought remains to be resolved and will without doubt receive more attention in the future. However, taken together, our data are consistent with NO under drought influencing the fate of N towards either the direct precursors of polyamines and polyamines themselves or away from these to other biosynthetic pathways including ethylene pathway, and leading to drought tolerance or susceptibility. Targeting of genes regulating the key biosynthetic steps revealed in our study would clearly be an attractive way of deriving genotypes which exhibited increased tolerance to water stress.

## Methods

### Plant Material

The derivation of the transgenic barley lines (*Hordeum vulgare* var. Golden Promise) expressing cDNA of the barley hemoglobin gene HvHb1 (accession number: U94968) controlled by the maize ubiquitin promoter and designated UHb has been described in^28^.

In line with previous literature, experiments were carried out at seedling stage^53–55^. Seedlings were grown in 0.5 L pots filled with peat: sand (3: 1) in a growth chamber at 20 °C, 65% relative humidity and under 12 h dark/12 h light periods with 250 μmol m^−2^ sec^−1^ photon flux density supplied by white fluorescent tubes (OSRAM). During growth, trays carrying the pots were watered regularly. When plants had two developed leaves and the third unrolled, water was withheld from those plants selected for drought treatment. Control plants were watered as described above throughout the whole experiment. During the drought treatment the relative water content of the soil was monitored daily and reached a level of approximately 20% by the end of the experiment (day 18).

### *In vivo* NO measurements in intact plants

NO production was measured using a quantum cascade laser (QCL)-based spectrometer, equipped with an astigmatic multipass absorption cell for wavelength modulation spectroscopy on NO^56^. For online concentration measurements and data analysis the LabVIEW program (National Instruments) was used^56^. The detector was calibrated using a certified calibration mixture with 100 ppbv NO (National Measurement Institute, Delft, The Netherlands). In each experiment four glass cuvettes (150 ml volume) containing one barley seedling each were measured. Soil and pots were autoclaved to prevent the growth of microorganisms that could modify the NO balance. NO was allowed to accumulate in the headspace for 75 min, and, thereafter, the cuvette was flushed with hydrocarbons-free air at a flow rate of 1.66 Lh^−1^, and accumulated NO was quantified. The four cuvettes were measured sequentially. One cuvette contained the well-watered WT plant, the second cuvette the well-watered UHb line, the third and fourth, contained respectively, droughted WT and UHb plants. For the following three replications on independent plants the order of the cuvettes was changed randomly.

### Visual assessment of drought symptoms

From the time at which water was withheld for drought treatment (from now on T0) all plants were visually evaluated daily according to the following scale: 0 = vigorous plant, with no leaves showing drought symptoms; 1 = one or two leaves (older leaves) show slight drought symptoms in the tips (less turgor) but most leaves remain erect; 2 = several leaves show a slight decrease in the turgor, however most of the leaves still show no drought symptoms; 3 = leaves show bending of the tip although the rest of the leaf remain turgid, incipient yellowing of the older leaf; 4 = all leaves show drought symptoms including incipient wilting and/or yellowing of the older leaf; 5 = all leaves start to appear rolled and or shrunken^33^. Five plants per accession were assessed. Drought severity values daily assessed according to this scale were used to calculate the area under the drought progress curve (AUDPC) for each accession similarly to the area under the disease progress curve widely used to disease screenings^57^ using the formula:

AUDPC = ∑ki = 1 ½ [(Si + Si + 1)(ti + 1 − ti)]

where Si is the drought severity at assessment date, ti is the number of days after the first observation on assessment date i and k is the number of successive observations carried out during the days after withholding water (daww). Measurements were performed on ten independent plants per genotype and treatment.

### Relative water content

Relative water content of leaves (lRWC) was measured in ten plants per genotype and treatment according to^58^. Measurements were carried out in the second leaves at 20% soil relative water content. Six hours after the onset of the light period, leaf blade segments were weighed (fresh weight; FW), floated on distilled water at 4 °C overnight and weighed again (turgid weight; TW). They were then dried at 80 °C for 48 h. After this, the dry weight (DW) was determined. RWC was then calculated as RWC = [(FW − DW)/(TW − DW)] × 100.

### Leaf water potential

Leaf water potential (*ψ*) was measured at midday with a pressure chamber (Soil Moisture Corp., Santa Barbara, CA, USA). Measurements were performed on ten independent plants per genotype and treatment.

### Transpiration assessment

Transpiration expressed in per leaf unit area was measured gravimetrically in 10 plants per genotype and treatment. The pots were covered from both ends with 2 polythene bags that were fixed to the pot with elastic bands. A small slit was made in the top of the bag to allow the plant to grow through. Control pots without plants showed minimum water loss. The initial and final (after each time point) pot weight was taken and transpiration was calculated by subtracting the final pot weight from the initial weight. Leaf area was calculated with software ImageJ after scanning the leaves fixed on sheets of paper.

### Polyamine quantification

The standard polyamines, putrescine (Put), spermidine (Spd), spermine (Spm) and 1–3, diaminopropane (DAP) were obtained as their hydrochlorides (Sigma) whereas agmatine (Agm) was obtained as its sulfate (Sigma). Leaves were fixed in liquid nitrogen and stored frozen until use. Plant extract were obtained by homogenizing the plant tissue in perchloric acid (0.1 w/v) according to^59^. Standards and plant extracts were benzoylated according to^60^. High performance liquid chromatography analysis of benzoyl-PAs was performed according to^61^, using an Agilent 2100 Series HPLC. 1, 7-diaminoheptane (HTD) was used as internal standard (Sigma).

### Amino acids quantification

Amino acids standards and the internal standard, norvaline were obtained from Sigma. Standard solutions were prepared from a stock solution by dilution with 0.1 M HCl. Leaves were fixed in liquid nitrogen and stored frozen until use. Plant extracts were obtained by homogenizing the plant tissue in 0.1 M HCl according to^62^. Analysis were carried out using an Agilent 2100 Series HPLC and a column Merck Lichro-CART®250-4 Superspher®100 RP-18 end-capped (25 cm × 4.6 mm; 5 mm particles) at 42 °C. Briefly, 40 µL of leaf extract was mixed with 200 µL borate buffer (0.4 M, pH 10.6), 200 µL OPA reagent and 40 µL FMOC reagent prepared according to^62^. The reaction mixture was allowed to stand for 2 min at 42 °C and then 20 µL was injected. The chromatographic separation was made using a binary gradient elution^62^. Mobile phase A was a 20 mM sodium acetate solution, with 0.018% (v/v) triethylamine, 0.3% (v/v) tetrahydrofurane, and 0.010% (v/v) of a 4% (m/v) solution of EDTA. The pH was adjusted to 7.20 with a 0.1% (v/v) solution of acetic acid. Mobile phase B was a solution with 20% (v/v) of a sodium acetate solution (100 mM, pH 6.0), 40% (v/v) of acetonitrile, 40% (v/v) of methanol, and 0.018% (v/v) triethylamine. Excitation/emission wavelengths were respectively 340/450 nm for primary amino acids and 237/340 nm for secondary amino acids. The latter was used to enhance the sensitivity of proline detection. The change in wavelengths was made at 115 min.

### Primer design

All primers used in this study (Table 1) were designed using the Universal Probe Library Assay Design Center (Roche applied Science) based on mRNA sequences deposited in GenBank. The specificity of the primers was checked by alignments with the original GenBank sequences using the standard nucleotide-nucleotide BLAST (blastn; provided online by NCBI http://blast.ncbi.nlm.nih.gov/Blast.cgi).

**Table 1:** Primers designed and used in real-time reverse transcription-polymerase chain reaction (qRT-PCR) for amplifying polyamine-associated genes of barley plants.

Target gene	Fw Primer	Rv Primer	Reference
HvHB1 (endogene)	TCGTCTTCAGCGAGGAGAAG	GATCTCGAAGATCTTGAGGAAG	69
Transgenic D-hordein:HvHB1	GATCAATTCATTGACAGTCCAC	TCGCAGGTCATGACGAAGAC	69
ADC	CATCATCGTGTTGGAGATGG	AGCTTGTTGCTCTGGTCGAT	70
ODC	CGGCTCCAACTTCAATGG	GTCAGCTGGAGTAGGCCAAG	71
MAT	CTTCACCAAGCGTCCAGAA	GCATCAGCTCAGGGGTCTC	71,72
AdoMetDC	GGCTCTCTCATCTACCAGAGCTT	GATCTTGGCGACCCACTG	71
ACS1	GTCTCCTCCCAGACGCAGTA	TGCGGGTGAAGTCCTTGT	73
ACS2	GAGTTCAGACAGGCGATGG	GTCAAACCTGGCCTTCCAC	73
ACS5	GAGCTGCTCACGTTCATCCT	CAAAACCCGGGTAGTACGG	73
ACS6	TCCTCCAGCTCTACATCAAGC	GAGGAGGAGGCCGAAGTG	73
GADPH	TGTCCATGCCATGACTGCAA	CCAGTGCTGCTTGGAATGATG	70

### RNA extraction and cDNA amplification

Total RNA was extracted from 100 mg of ground leaf tissue using previously reported protocols^63,64^. RNA was purified using RNeasy® Minelute Cleanup Kit (QIAGEN). Removal of any residual genomic DNA in RNA samples was verified by PCR amplification of total RNA (with no cDNA synthesis step) using the designed primers listed in Table 1. RNA samples containing DNA were further DNase treated until no PCR amplification of RNA samples was obtained. Prior to retrotranscription the concentration and integrity of RNA were verified by an optical density at 260 nm (OD260)/OD280 absorption ratio in a NanoDrop ND- 1000 spectrophotometer (Thermo scientific).

First and second-strand of complementary DNA (cDNA) were synthesized using SuperScript® III First-Strand (Invitrogen) and DNA Polymerase I (BioLabs), respectively. cDNA was cleaning by QUIquick PCR Purification Kit (QIAGEN and DNase treated by the RNase-Free DNase Set (Qiagen), according to the manufacturer’s recommendations. Conventional RT-PCR and PCR assays followed by gel electrophoresis were performed to verify the amplification of cDNA using the designed primers. Quality and quantity of cDNA was determined by running aliquots in agarose gels and by spectrophotometric analysis in a NanoDrop ND-1000 spectrophotometer (Thermo scientific).

### Gene expression analysis by real-time RT-PCR

Previous to test the expression of the polyamine-associated genes, four additional genes were tested for using as reference genes; glyceraldehyde-3-phosphate dehydrogenase (GADPH), beta-tubulin (TUBB), alpha-tubulin (TUBA) and 18 S ribosomal RNA (18 S rRNA) according to^65^. Following preliminary assay, GADPH was selected as internal control since it showed a highly stable expression in our barley samples. Real-time qRT-PCR was performed for each of the polyamine-associated genes and for GADPH on at least 3 independent biological plus 3 technical replicated cDNA templates in StepOne Real-Time PCR System (Applied Biosystems) using FastStart Universal SYBR Green Master (Rox) (Roche) according to the manufacturer’s recommendations. The reaction mixture contained 10 μl of SYBR Green master mix, 6 μl of each primer set (Table 1) and 4 μl of cDNA or standard solution as template. The amplification conditions were 95 °C for 10 min, followed by 40 cycles of amplification at 95 °C for 15 s, 60 °C for 1 min. Following amplification, a melting curve program 95 °C for 15 s, 60 °C for 1 min and 60 to 95 °C at a heating rate of 0.3 °C/min was used. The melting point analysis was performed at the end of the real-time qRT-PCR to confirm the amplification of a unique product for each gene. The fold changes of polyamine-associated gene transcripts in different treatments versus control (i.e. well-watered plants) were normalized using the CT and efficiency obtained for the GADPH amplification run on the same cDNA templates according to the 2^−∆∆Ct^ method^66^.

### *In vivo* ethylene measurements in intact plants

Ethylene production was monitored in real time using a gas flow-through in-line system fitted with a laser-based photoacoustic ethylene detector (ETD-300, Sensor Sense, the Netherlands), which is able to detect on-line 300 parts per trillion per volume of ethylene within 5 s^67^. Ethylene emanation from a single seedling placed into a glass cuvette (254 ml volume) was alternately monitored for 15 min (5 s per acquisition point), at a controlled continuous flow rate of 1.5 l h^−1^ by flushing with air and preventing accumulation-induced effects. Both the gas flow and the alternative switching between cuvettes was regulated by an automated valve control box (VC-6, Sensor Sense, the Netherlands). KOH and CaCl_2_ scrubbers were incorporated into the gas flushing system to remove CO_2_ and H_2_O respectively, before entering the ethylene detector. As assays of ethylene production from barley plants inoculated with B. cinerea involved measuring both the plant and the compost, a reference cuvette was included containing only a module of compost. The negligible level of ethylene produced from this compost was subtracted from plant cuvettes.

### Leaf chlorophyll content

Leaf chlorophyll was indirectly estimated on the first leaves of five plants per accession, using a SPAD-502 chlorophyll meter (Minolta Co., LTD., Japan)^68^. The final measurement of each plant was the mean of three measurements per leaf, the adaxial side of the leaves was always placed toward the emitting window of the instrument. Measurements were taken 6 hours after the onset of the light period at 6, 9, 12, 15 and 18 daww in control and stressed plants.

### Statistical analysis

For the different experiments, at least four leaves, each from a different plant per genotype and treatment were studied in completely randomized designs. For statistical analysis, data recorded as percentages were transformed to arcsine square roots (transformed value = 180/п x arcsine [√(%/100)]) to normalize data and stabilize variances throughout the data range. However, for ease of understanding means of raw percentage data are presented in figures. Data were subjected to ANOVA analysis of variance using SPSS software and residual plots were inspected to confirm data conformed to normality. Significance of differences between means was determined by contrast analysis (Scheffe’s).

## Electronic supplementary material

Supplementary information

## References

[1] Farooq M, Wahid A, Kobayashi N, Fujita D, Basra SMA (2009). Plant drought stress: effects, mechanisms and management. Agron. Sustain. Dev..

[2] Bandurska H, Niedziela J, Chadzinikolau T (2013). Separate and combined responses to water deficit and UV-B radiation. Plant Sci..

[3] Mur LAJ (2012). Haemoglobin modulates salicylate and jasmonate/ethylene-mediated resistance mechanisms against pathogens. J. Exp. Bot..

[4] Wendehenne D, Pugin A, Klessig DF, Durner J (2001). Nitric oxide: comparative synthesis and signaling in animal and plant cells. Trends Plant Sci..

[5] Hancock, J. T., Lisjak, M., Teklic, T., Wilson, I. D. & Whiteman, M. Hydrogen sulfide and signaling in plants. *CAB Reviews*, 6–12 (2011). 10.4161/psb.6.10.17104 PMC3256366 21904118

[6] Bright J, Desikan R, Hancock JT, Weir IS, Neill SJ (2006). ABA-induced NO generation and stomatal closure in Arabidopsis are dependent on H2O2 synthesis. Plant J..

[7] Neill SJ, Desikan R, Clarke A, Hancock JT (2002). Nitric oxide is a novel component of abscisic acid signaling in stomatal guard cells. Plant Physiol..

[8] Garcia-Mata C, Lamattina L (2001). Nitric oxide induces stomatal closure and enhances the adaptive plant responses against drought stress. Plant Physiol..

[9] Ribeiro RV, Machado EC, Santos MG, Oliveira RF (2009). Photosynthesis and water relations of well-watered orange plants as affected by winter and summer conditions. Photosynthetica.

[10] Bachrach U (2005). Naturally occurring polyamines: Interaction with macromolecules. Curr Protein Pept Sci..

[11] Cohen AR (1998). Human CASK/LIN-2 binds syndecan-2 and protein 4.1 and localizes to the basolateral membrane of epithelial cells. J. Cell Biol..

[12] Kuznetsov VV, Shevyakova NI (2007). Polyamines and stress tolerance of plants. Plant Stress.

[13] Martin-Tanguy J (1997). Conjugated polyamines and reproductive development: Biochemical, molecular and physiological approaches. Physiol. Plant..

[14] Tun NN (2006). Polyamines induce rapid biosynthesis of nitric oxide (NO) in Arabidopsis thaliana seedlings. Plant Cell Physiol..

[15] Yamaguchi K (2007). A protective role for the polyamine spermine against drought stress in Arabidopsis. Biochem. Biophys. Res. Co..

[16] Turner LB, Stewart GR (1986). The effect of water-stress upon polyamine levels in barley (*Hordeum vulgare* L.) leaves. J. Exp. Bot..

[17] Yang P, Chen H, Liang Y, Shen S (2007). Proteomic analysis of de-etiolated rice seedlings upon exposure to light. Proteomics.

[18] Gao C, Hu J, Zhang S, Zheng Y, Knapp A (2009). Association of polyamines in governing the chilling sensitivity of maize genotypes. Plant Growth Regul..

[19] Wu GY, Morris SM (1998). Arginine metabolism: nitric oxide and beyond. Biochemical Journal.

[20] Flores T (2008). Arginase-negative mutants of Arabidopsis exhibit increased nitric oxide signaling in root development. Plant Physiol..

[21] Freschi, F. Nitric oxide and phytohormone interactions: current status and perspectives. *Plant Sci*., 10.3389/fpls.2013.00398 (2013). 10.3389/fpls.2013.00398 PMC3793198 24130567

[22] Fan H-F, Du C-X, Guo S-R (2013). Nitric oxide enhances salt tolerance in cucumber seedlings by regulating free polyamine content. Environ Exper Bot..

[23] Arasimowicz-Jelonek M, Floryszak-Wieczorek J, Kubis J (2009). Interaction Between Polyamine and Nitric Oxide Signaling in Adaptive Responses to Drought in Cucumber. J Plant Growth Regul..

[24] Igamberdiev AU, Bykova NV, Hill RD (2011). Structural and Functional Properties of Class 1 Plant Hemoglobins. Iubmb Life.

[25] Hebelstrup KH, Shahb JK, Igamberdiev AU (2013). The role of nitric oxide and hemoglobin in plant development and morphogenesis. Physiol. Plant..

[26] Hill, R. D. Non-symbiotic haemoglobins-What’s happening beyond nitric oxide scavenging? *Aob Plants*, 10.1093/aobpla/pls004 (2012). 10.1093/aobpla/pls004 PMC3292739 22479675

[27] Hebelstrup KH (2012). Haemoglobin modulates NO emission and hyponasty under hypoxia-related stress in *Arabidopsis thaliana*. J. Exp. Bot..

[28] Hebelstrup KH (2014). An assessment of the biotechnological use of hemoglobin modulation in cereals. Physiol. Plant..

[29] Gupta KJ, Fernie AR, Kaiser WM, van Dongen JT (2011). On the origins of nitric oxide. Trends Plant Sci..

[30] Alcázar R (2010). Polyamines: molecules with regulatory functions in plant abiotic stress tolerance. Planta.

[31] Abeles, F. B., Morgan, P. W. & Saltveit, M. E. *Ethylen in plant biology*. 414 (1992).

[32] Neill SJ, Desikan R, Hancock JT (2003). Nitric oxide signalling in plants. New Phytologist.

[33] Sánchez-Martín J (2015). A metabolomic study in oats (*Avena sativa)* highlights a drought tolerance mechanism based upon salicylate signalling pathways and the modulation of carbon, antioxidant and photo-oxidative metabolism. Plant Cell Environ..

[34] Steudle E (2000). Water uptake by roots: effects of water deficit. J. Exp. Bot..

[35] Hebelstrup KH, Jensen EO (2008). Expression of NO scavenging hemoglobin is involved in the timing of bolting in Arabidopsis thaliana. Planta.

[36] de Andres, M. C., Maneiro, E., Martin, M. A., Arenas, J. & Blanco, F. J. Nitric oxide compounds have different effects profiles on human articular chondrocyte metabolism. Arthritis Research & Therapy **15**, 10.1186/ar4295 (2013). 10.1186/ar4295 PMC3978712 24025112

[37] Walters DR (2000). Polyamines in plant-microbe interactions. Physiol. Mol. Plant Pathol..

[38] Galston AW, KaurSawhney R, Altabella T, Tiburcio AF (1997). Plant polyamines in reproductive activity and response to abiotic stress. Bot. Acta.

[39] Malmberg RL, Cellino ML (1994). Arginine decarboxylase of oat is activated by enzymatic cleavage into two polypeptides. J. Biol. Chem..

[40] Malmberg RL, Smith KE, Bell E, Cellino ML (1992). Arginine decarboxylase of oats is clipped from a precursor into 2-polypeptides found in the soluble enzyme. Plant Physiol..

[41] Capell T (1998). Over-expression of the oat arginine decarboxylase cDNA in transgenic rice (Oryza sativa L.) affects normal development patterns *in vitro* and results in putrescine accumulation in transgenic plants. Theor Appl Genet..

[42] Capell T, Bassie L, Christou P (2004). Modulation of the polyamine biosynthetic pathway in transgenic rice confers tolerance to drought stress. Proc. Natl. Acad. Sci. USA..

[43] Pegg AE, Xiong H, Feith DJ, Shantz LM (1998). S-adenosylmethionine decarboxylase: structure, function and regulation by polyamines. Biochem. Soc. Trans..

[44] Hillary RA, Pegg AE (2003). Decarboxylases involved in polyamine biosynthesis and their inactivation by nitric oxide. BBA-Proteins Proteom.

[45] Apelbaum A, Yang SF (1981). Biosynthesis of stress ethylene induced by water deficit. Plant Physiol..

[46] Young TE, Meeley RB, Gallie DR (2004). ACC synthase expression regulates leaf performance and drought tolerance in maize. Plant J..

[47] Ederli L (2006). Interaction between nitric oxide and ethylene in the induction of alternative oxidase in ozone-treated tobacco plants. Plant Physiol..

[48] Mur LAJ, Laarhoven LJJ, Harren FJM, Hall MA, Smith AR (2008). Nitric Oxide Interacts with Salicylate to Regulate Biphasic Ethylene Production during the Hypersensitive Response. Plant Physiol..

[49] Garcia MJ, Suarez V, Romera FJ, Alcantara E, Perez-Vicente R (2011). A new model involving ethylene, nitric oxide and Fe to explain the regulation of Fe-acquisition genes in Strategy I plants. Plant Physiol Biochem..

[50] Barbosa, F. *et al*. Implications of ethylene biosynthesis and signaling in soybean drought stress tolerance. *BMC Plant Biol*. **15**, 10.1186/s12870-015-0597-z (2015). 10.1186/s12870-015-0597-z PMC4557918 26335593

[51] Van de Poel, B. & Van Der Straeten, D. 1-aminocyclopropane-1-carboxylic acid (ACC) in plants: more than just the precursor of ethylene! *Front Plant Sci*. **5**, 10.3389/fpls.2014.00640 (2014). 10.3389/fpls.2014.00640 PMC4227472 25426135

[52] Yoon, G. M. & Kieber, J. J. 1-Aminocyclopropane-1-carboxylic acid as a signalling molecule in plants. *Aob Plants* **5**, 10.1093/aobpla/plt017 (2013).

[53] Gong DS (2010). Early activation of plasma membrane H + -ATPase and its relation to drought adaptation in two contrasting oat (Avena sativa L.) genotypes. Environ Exper Bot..

[54] Hao Z (2010). Meta-analysis of constitutive and adaptive QTL for drought tolerance in maize. Euphytica.

[55] Sánchez-Martín J, Rubiales D, Sillero J, Prats E (2012). Identification and characterization of sources of resistance in *Avena sativa*, *A. byzantina* and *A. strigosa* germplasm against a pathotype of *Puccinia coronata* f.sp *avenae* with virulence against the Pc94 resistance gene. Plant Pathol..

[56] Cristescu SM, Persijn ST, Hekkert STL, Harren FJM (2008). Laser-based systems for trace gas detection in life sciences. Applied Physics B-Lasers and Optics.

[57] Jeger MJ, Viljanen-Rollinson SLH (2001). The use of the area under the disease-progress curve (AUDPC) to assess quantitative disease resistance in crop cultivars. Theor Appl Genet..

[58] Barrs HD, Weatherley PE (1962). A re-examination of the relative turgidity technique for estimating water deficits in leaves. Aust. J. Biol. Sci..

[59] Flores HE, Galston AW (1982). Analysis of polyamines in higher-plants by high-performance liquid-chromatography. Plant Physiol..

[60] Redmond JW, Tseng A (1979). High-pressure liquid chromatographic determination of putrescine, cadaverine, spermidine and spermine. J. Chromatogr..

[61] Slocum RD, Flores HE, Galston AW, Weinstein LH (1989). Improved method for HPLC analysis of polyamines, agmatine and aromatic monoamines in plant-tissue. Plant Physiol..

[62] Herbert P, Barros P, Ratola N, Alves A (2000). HPLC determination of amino acids in musts and port wine using OPA/FMOC derivatives. J. Food Sci..

[63] Chomczynski P, Sacchi N (1987). Single-step method of RNA isolation by acid guanidinium thiocyanate-phenol-chloroform extraction. Anal. Biochem..

[64] Raeder, U. & Broda, P. in *Letters in Applied Microbiology* Vol. 1 17–20 (1985).

[65] Jarosova, J. & Kundu, J. K. Validation of reference genes as internal control for studying viral infections in cereals by quantitative real-time RT-PCR. *BMC Plant Biol*. **10**, 10.1186/1471-2229-10-146 (2010). 10.1186/1471-2229-10-146 PMC3095291 20630112

[66] Livak KJ, Schmittgen TD (2001). Analysis of relative gene expression data using real-time quantitative PCR and the 2(T)(-Delta Delta C) method. Methods.

[67] Cristescu SM (2013). Current methods for detecting ethylene in plants. Annals of Botany.

[68] Sanchez-Martin J, Rubiales D, Sillero JC, Prats E (2012). Identification and characterization of sources of resistance in *Avena sativa*, *A. byzantina* and *A. strigosa* germplasm against a pathotype of *Puccinia coronata* f.sp *avenae* with virulence against the Pc94 resistance gene. Plant Pathol..

[69] Hebelstrup, K. H. *et al*. UCE: A uracil excision (USER (TM))-based toolbox for transformation of cereals. *Plant Methods* **6**, 10.1186/1746-4811-6-15 (2010). 10.1186/1746-4811-6-15 PMC2892451 20537147

[70] Sato K (2009). Development of 5006 Full-Length CDNAs in Barley: A Tool for Accessing Cereal Genomics Resources. DNA Res..

[71] Matsumoto T (2011). Comprehensive Sequence Analysis of 24,783 Barley Full-Length cDNAs Derived from 12 Clone Libraries. Plant Physiol..

[72] Mori, S. & Takizawa, R. *Barleysam1 gene* (2006).

[73] Dahleen LS, Tyagi N, Bregitzer P, Brown RH, Morgan WC (2012). Developing tools for investigating the multiple roles of ethylene: identification and mapping genes for ethylene biosynthesis and reception in barley. Mol Genet Genomics.

