# Supplementary material for: Reduced nitric oxide levels during drought stress promote drought tolerance in barley and is associated with elevated polyamine biosynthesis
Source: Sci Rep. 2017 Oct 17;7:13311. doi: 10.1038/s41598-017-13458-1 (PMC5645388; doi:10.1038/s41598-017-13458-1)
Supplement: Supplementary file 1 — Supplementary information [file 41598_2017_13458_MOESM1_ESM.pdf]

Supplementary Information

Reduced nitric oxide levels during drought stress promote drought tolerance in barley and is associated with elevated polyamine biosynthesis.

Gracia Montilla-Bascón[1], Diego Rubiales[1], Kim H Hebelstrup[2], Julien Mandon[3], Frans J.M. Harren[3], Simona M. Cristescu[3], Luis AJ Mur[4], and Elena Prats[1]*

[1]CSIC, Institute for Sustainable Agriculture, Córdoba, Spain.

[2]Section of Crop Genetics and Biotechnology, Department of Molecular Biology and Genetics Aarhus University, Slagelse, Denmark

[3]Radboud University, Department of Molecular and Laser Physics. Nijmegen, the Netherlands

[4]Institute of Biological, Environmental and Rural Sciences, University of Aberystwyth, UK

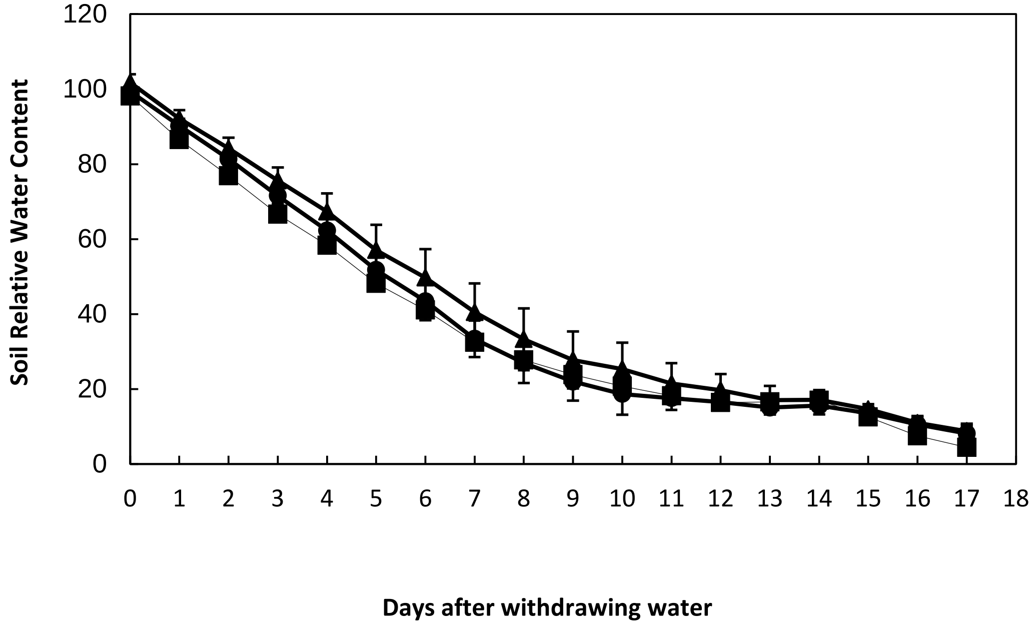

Supplementary Figure 1 . Soil relative water content in WT (circles) and transgenic UHb-05 (triangles) and UHb-06 (squares) plants during a time course of drought. Data are mean of two independent experiments, each with ten replicates per genotypes. Bars indicates standars errors.

Supplementary Table 1. Pearson correlations between total polyamines (PA) and their most direct amino acids precursors (Arginine, ARG; Citrulline, CIT; Methionine, MET; Ornithine, ORN) and PA derivatives (ϒ-aminobutiric acid, GABA; and 1,3-diaminopropane, DAP) in barley WT and UHb plants. *, **, and *** indicate significant differences at $P<0,05$; 0,01 and 0,001 respectively

WT							
**ARG**							
**CIT**	0.0623						
**DAP**	0.3337	-0.7226*					
**GABA**	-0.5382	-0.7055	0.3477				
**MET**	0.1666	-0.2348	0.3625	-0.2059			
**ORN**	-0.4259	-0.8659**	0.4871	0.8505**	0.0917		
**Total PA**	0.1160	-0.8352**	0.7566*	0.6271	0.4031	0.7241*	
UHb							
**ARG**							
**CIT**	-0.8311*						
**DAP**	0.8441**	-0.6661					
**GABA**	-0.8567**	0.6939	-0.7774*				
**MET**	0.6942	-0.5999	0.8253*	-0.8705**			
**ORN**	0.8117*	-0.6358	0.8997**	-0.8191*	0.8854**		
**Total PA**	0.8209*	-0.5433	0.9711***	-0.8056*	0.8137*	0.8926**	
	**ARG**	**CIT**	**DAP**	**GABA**	**MET**	**ORN**	**Total PA**
